# Adaptation to public goods cheats in *Pseudomonas aeruginosa*

**DOI:** 10.1098/rspb.2017.1089

**Published:** 2017-07-26

**Authors:** Siobhán O'Brien, Adela M. Luján, Steve Paterson, Michael A. Cant, Angus Buckling

**Affiliations:** 1 Center for Adaptation to a Changing Environment (ACE), ETH Zürich, 8092 Zürich, Switzerland; 2 Centro de Investigaciones en Química Biológica de Córdoba, CIQUIBIC, CONICET and Departamento de Química Biológica, Facultad de Ciencias Químicas, Universidad Nacional de Córdoba, Ciudad Universitaria, X5000HUA Córdoba, Argentina; 3 Institute of Integrative Biology, University of Liverpool, Crown Street, Liverpool L69 7ZB, UK; 4 College of Life and Environmental Sciences, University of Exeter, Penryn Campus, Cornwall TR10 9FE, UK; 5 Environment and Sustainability Institute, University of Exeter, Penryn Campus, Cornwall TR10 9FE, UK

**Keywords:** siderophore, public goods, cooperation, *Pseudomonas*, pyoverdine, experimental evolution

## Abstract

Cooperation in nature is ubiquitous, but is susceptible to social cheats who pay little or no cost of cooperation yet reap the benefits. The effect such cheats have on reducing population productivity suggests that there is selection for cooperators to mitigate the adverse effects of cheats. While mechanisms have been elucidated for scenarios involving a direct association between producer and cooperative product, it is less clear how cooperators may suppress cheating in an anonymous public goods scenario, where cheats cannot be directly identified. Here, we investigate the real-time evolutionary response of cooperators to cheats when cooperation is mediated by a diffusible public good: the production of iron-scavenging siderophores by *Pseudomonas aeruginosa*. We find that siderophore producers evolved in the presence of a high frequency of non-producing cheats were fitter in the presence of cheats, at no obvious cost to population productivity. A novel morphotype independently evolved and reached higher frequencies in cheat-adapted versus control populations, exhibiting reduced siderophore production but increased production of pyocyanin—an extracellular toxin that can also increase the availability of soluble iron. This suggests that cooperators may have mitigated the negative effects of cheats by downregulating siderophore production and upregulating an alternative iron-acquisition public good. More generally, the study emphasizes that cooperating organisms can rapidly adapt to the presence of anonymous cheats without necessarily incurring fitness costs in the environment they evolve in.

## 1. Background

Cooperative behaviour (any action selected at least partly because of its beneficial effect on another individual [1]) owes its ubiquity to incurring a direct or indirect fitness benefit to individuals in cooperating groups. However, when cooperation carries a cost, it is associated with social cheats who pay little or no cost of cooperation but reap the rewards. This fitness advantage facilitates their invasion of cooperating groups, which can impose a large cost on the population as a whole, in a ‘Tragedy of the Commons' scenario [2,3]. However, cooperation persists at all levels of biological organization [4], suggesting that mechanisms have evolved that impede the negative impact of cheats. Many organisms, including long-tailed tits [5], bumblebees [6] and toads [7,8], preferentially direct help towards relatives, while tactics such as punishing/policing cheats have been well documented, for example, in queenless ants [9], honeybees [10] and humans [11,12].

Our understanding of how cooperators adapt to the presence of cheats has been greatly enhanced by studies of microbes, owing to their suitability for carrying out real-time evolution experiments as well as identifying the genetic basis of behaviours. Maintaining high relatedness between producer and beneficiary is paramount for allowing cooperation to persist [13], so that directing the benefits of cooperation to close kin or clone mates can mitigate the negative influence of cheats on population growth [14]. Preferential interactions with close relatives can be facilitated by a spatially structured environment [15], but even in the absence of spatial structure mechanisms such as ‘green beard’ genes [16,17] and antagonistic pleiotropy [18,19] facilitate directing the benefits of cooperation to kin.

Several studies have demonstrated real-time evolution of cheat resistance in cooperator populations. *Pseudomonas fluorescens* biofilms can be invaded by non-contributing cheats, compromising the integrity of the biofilm. Coevolution between these two phenotypes gives rise to increasingly efficient cheats and more resistant cooperators [20]. Moreover, cooperators have been found to evolve ways of opposing productivity of cheats who fail to contribute to dead-end stalk cells in *Myxococcus xanthus* [21], and *Dictyostelium discoideum* [22]. However, the exact mechanism of cheat resistance in these cases is unclear.

While such examples involve direct physical association between producer and cooperative trait, it is less clear if and how adaptation may occur in an anonymous public good scenario, where there is usually no clear physical link between producer and product. Hence, directly targeting cheats for punishment/policing or directing benefits of cooperation towards kin is problematic. Moreover, simple point mutations often lead to the rapid production of cheats, making pleiotropy unlikely as an anti-cheating mechanism [23]. Several recent studies have suggested novel ways by which bacteria can fine-tune their cooperative output to ensure beneficiaries are highly related. For instance, quorum sensing can be used by bacteria to infer when they are surrounded by clone mates, allowing them to tune their investment into cooperative traits depending on the genotype of surrounding cells [24,25]. Similarly, *Escherichia coli* plasmid donors can bias altruistic transfer of beneficial plasmids only to other cells that share the donation alleles [26]. However, this requires a degree of population structuring or an association between transfer and discrimination alleles.

Here, we investigate the real-time evolutionary response of cooperators to cheats when cooperation is mediated by a public good that is individually costly and carries a group-level benefit: the production of iron-scavenging siderophores by *Pseudomonas aeruginosa*. Cheats evolve rapidly in this system, avoiding the cost of siderophore production while still retaining the correct receptor for uptake of the siderophore–iron complex [1,27]. Two recent studies coevolved siderophore producers and cheats together, and reported the evolution of reduced siderophore production in producer populations [28,29]. However, in these studies, either the fitness consequences of altered siderophore production were not assessed [28] or the experimental design did not impose selection for cooperation, so that cheating, rather than resistance to cheating, was selected for [29]. As such, these studies did not determine whether or not cooperators can adapt to cheats. Here, we evolve *P. aeruginosa* in the presence or the absence of cheats, under conditions where there is selection for cooperation: patches within a metapopulation are mixed and single cooperator clones from this mixture used to inoculate new patches. Mixing patches means that genotypes from the most productive patches, i.e. in which cooperators are less exploited by cheats, are over-represented in subsequent generations, while those from less productive patches are under-represented. Inoculating new patches with single clones resulted in high relatedness, and hence stronger selection for cooperation [30].

## 2. Material and methods

### (a) Strains and growth media

The *P. aeruginosa* strain PAO1 was used as the siderophore-producing wild-type. A gentamicin-resistant PAO1 (PAO1^R^) and gentamicin-resistant PAO1 with a *lacZ* reporter gene insertion (PAO1^R^*lacZ*) were engineered by integrating a gentamicin resistance cassette (Tn7-gm) and a *lacZ* gene (with a gentamicin resistance cassette; Tn7-gm-*lacZ*), respectively, at the *att*::Tn7 locus in *P. aeruginosa* PAO1 [31]. PAO1*ΔpvdDΔpchEF* is a gentamicin-susceptible isogenic mutant strain of PAO1 with genes encoding both primary and secondary siderophores, pyoverdine and pyochelin knocked out [32]. Experiments were carried out in Kings Medium B (KB) [33]: (10 g glycerol, 20 g proteose peptone no. 3, 1.5 g K_2_HPO_4_·3H_2_O, 1.5 g MgSO_4_·7H_2_O per litre). Where stated, KB medium was made iron-limited by the addition of freshly made filter-sterilized 100 µg ml^−1^ human apotransferrin and 20 mM NaHCO_3_ to KB medium immediately before use. As siderophore production is repressed when there is an excess of Fe^2+^ [34]; iron-limitation ensures that siderophores are essential for growth and stimulates their production. Gentamicin was used at a concentration of 30 µg ml^−1^ and 5-Bromo-4-chloro-3-indolyl-β-d-galactopyranoside (X-gal) at 90 µg ml^−1^. Bacteria were grown at 37°C shaken at 180 r.p.m. unless stated otherwise.

### (b) Costs and benefits of siderophore production

We firstly confirmed that siderophore production carries a cost, and is exploitable by non-producing cheats in our experimental context: 6 ml iron-limited shaken KB medium. We established six PAO1 populations (cooperator), six PAO1*ΔpvdDΔpchEF* populations (cheat) and six populations in 1 : 1 co-culture, quantifying relative fitness of cheats after 24 h. Proportions of bacteria were inoculated, so that the density of bacteria added to each microcosm was approximately 10^7^ colony forming units (CFUs) ml^−1^. Bacterial densities were assessed by plating appropriate dilutions on KB agar after 24 h growth.

### (c) Evolution experiment

We evolved cooperators (PAO1^R^) in the presence and absence of a high frequency (90%) of siderophore-negative cheats (PAO1*ΔpvdDΔpchEF*) (electronic supplementary material, figure SA1). This high frequency of cheats ensures there is selection for cooperators to adapt to mitigate the adverse effects of cheats on population productivity. Our design comprised six replicate populations for each treatment (±cheats), with each population consisting of six ‘patches’. Patches were initiated with a single cooperator colony; 10^7^ CFUs ml^−1^ for the control treatment and 10^6^ CFUs ml^−1^ for the 90% cheat treatment. A total of 9 × 10^6^ CFUs ml^−1^ PAO1*ΔpvdDΔpchEF* were also added to the 90% cheat treatment, so that the final inoculated bacterial density was also 10^7^ CFUs ml^−1^. After 24 h growth, 100 µl was combined from each patch and plated on KB agar + gentamicin (facilitating cheat removal). Single colonies were then selected at random to inoculate new patches, and cheats were re-added to the appropriate treatment, using the same number of CFUs as before. This design (global competition and high relatedness) selected for subpopulations with high productivity, because genotypes from the most productive subpopulations were over-represented in the mixture (see [18]). This process of enforcing global competition was repeated 18 times (approx. 190 generations), but cultures were allowed to grow for 96 h (rather than 24 h) between the enforcement of the final four rounds of global competition, transferring 1% of the cells to fresh media every 24 h. This was to accelerate evolutionary change which we speculated was being constrained by daily bottlenecking of cultures.

### (d) Quantifying fitness of evolved populations: competition experiments

Fitness of each of our 12 evolved populations was assessed relative to their ancestor in both selective environments: in the presence and absence of cheats. Six replicates were established per population in 6 ml Fe-limited medium, totalling 144 fitness assays. Half of the tubes were inoculated with approximately 5 × 10^5^ CFUs ml^−1^ ancestral PAO1^R^*lacZ* (gentamicin-resistant and *lacZ* insertion strain), approximately 5×10^5^ CFU's ml^−1^ of the appropriate evolved population and approximately 9 × 10^6^ CFUs ml^−1^ of PAO1*ΔpvdDΔpchEF*, so that cheat strains represented a high proportion (approx. 90%) of the total bacterial density in each microcosm, and the total inoculated density was 10^7^ CFU's ml^−1^. A further 72 microcosms (cheat-free competitions) were inoculated with approximately 5 × 10^6^ CFU's ml^−1^ ancestral PAO1^R^*lacZ* and approximately 5 × 10^6^ CFU's ml^−1^ of the appropriate evolved population, so the total inoculum was 10^7^ CFUs ml^−1^. Microcosms were grown for 24 h, after which densities were assessed by plating liquid cultures on KB agar supplemented with 30 µg ml^−1^ gentamicin and 90 µg ml^−1^ Xgal, and counting viable colonies. Gentamicin facilitated the removal of cheats at the counting stage, which otherwise would have dominated the plates and resulted in very low cooperator counts. Evolved and ancestral cooperator strains were distinguished by a dark blue appearance of the ancestral strain on Xgal-supplemented agar. Finally, the neutrality of the *lacZ* insertion in the ancestral strain under these growth conditions was confirmed by competing PAO1^R^*lacZ* with PAO1^R^ at 1 : 1 in Fe-limited KB media.

### (e) Measuring public goods production

After approximately 190 generations, each replicate was diluted and cultured on KB agar to measure: (i) colony morphotypic variation; (ii) *per capita* siderophore production; (iii) production of the most costly and efficient iron–siderophore, pyoverdine [35–37]; and (iv) the toxin pyocyanin, which can generate soluble iron.

Thirty randomly selected colonies from each population were statically grown in 200 µl iron-limited KB medium (siderophore-stimulating conditions). *Per capita* total siderophore production was quantified by combining 50 µl from each of the 30 single colony cultures, centrifuging to pellet cells and performing a 50% Chrome azurol S (CAS) assay on the supernatant, measuring *A*_630_ of cultures as well as the cell-free supernatant (reference culture) [38,39]. A measure of iron chelator activity relative to the reference culture in each population was given by [1 − (*A*_pop_/*A*_ref_)], standardized by the optical density (OD; *A*_600_) of the relevant culture. *Per capita* pyoverdine was quantified for each of our 30 isolated colonies per population using a pyoverdine-specific emission assay [40]. Briefly, fluorescence of each culture was measured at 460 nm following excitation at 400 nm, using a Biotek Synergy 2 Spectrophotometer. OD was measured at 600 nm, and the ratio of relative fluorescence units (RFUs)/OD was employed as a quantitative measure of *per capita* pyoverdine production. Finally, evolved populations were analysed for production of the toxin pyocyanin, which can promote soluble ferrous iron [41]. Briefly, all evolved populations were plated on gentamicin-supplemented agar to remove cheats. Wild-type cells (with gentamicin resistance) were washed from plates using 6 ml KB broth, and grown overnight in 30 ml glass tubes. After 24 h, cells were centrifuged, and *A*_691_ was measured for each population, standardized by *A*_600_. Ancestral wild-type, cheat and uninoculated KB broth were included in all of our assays as controls.

### (f) Quantifying evolved population productivity

To investigate whether any adaptation to cheats in treatment populations sacrificed population productivity, the relative growth rate of each evolved population was assessed by growing each evolved population for 24 h in Fe-limited KB media. Final densities were quantified by plating liquid cultures on KB agar and counting viable colonies.

### (g) Addition of exogenous pyocyanin

We investigated whether pyocyanin could rescue the poor fitness of siderophore cheats in an iron-limited environment. We tested the effect of 10, 30 and 50 µM pyocyanin on the growth of both PAO1 (cooperator) and siderophore-negative cheat PAO1*ΔpvdDΔpchEF*, relative to a pyocyanin-free control. 10^7^ CFUs ml^−1^ of cooperator or cheat was inoculated into 6 ml iron-limited pyocyanin-supplemented KB and grown for 24 h. Final densities were assessed by plating on KB agar.

### (h) Resequencing methods and bioinformatic analysis

The Wizard^®^ Genomic DNA Purification kit (Promega) was used to isolated genomic DNA from overnight cultures, according to the manufacturer's instructions. The quality of the isolated gDNA was assessed using Nanodrop (Thermo Scientific). Four smooth and four novel morphotypes from endpoint cheat-adapted populations were selected for sequencing. TruSeq PCR-free genomic libraries were prepared at the Centre for Genomic Research, University of Liverpool and 2 × 250 bp paired-end reads generated on an Illumina MiSeq platform. See the electronic supplementary material, SA2 for further details on sequence data preparation.

### (i) Statistical analyses

All data were analysed using R v. 2.15.1 [42]. We determined population Malthusian growth rate (*m*) as ln(final density/start density) [43]. Relative fitness of strain *x* compared with strain *y* (*W*(*x*)) was calculated in co-culture as *m*(strain *x*)/*m*(strain *y*), and in monoculture as *m*(strain *x*)/mean(*m*(strain *y*)). When *W*(*x*) = 1, fitness of strain *x* = strain *y.* Following an *F*-test to compare variances, and a Shapiro–Wilk normality test, we used Student's *t*-tests and Wilcoxon rank-sum tests to compare *m* values in monoculture, or *W* values between treatments. To assess whether (*W*(*x*)) was significantly different from 1, we used one-sample *t*-tests or Wilcoxon signed-rank tests.

One- and two-sample *t*-tests were used to compare *per capita* total siderophore production between evolved populations and between evolved populations and the ancestor (using mean ancestral siderophore production as the alternative value in one-sample tests), and a Kolmogorov–Smirnov test for non-parametric data with unequal variances was employed to compare the frequency of a novel morphotype in control and treatment populations. A one-sample *t*-test and Wilcoxon signed-rank were used to assess whether evolved pyoverdine production in control and treatment populations differed significantly from that of the ancestor. To compare *per capita* pyoverdine between evolved populations, we used a linear mixed effects revised (LMER) model, assigning condition (treatment/control) as a fixed factor and population as a random factor, controlling for the presence of 30 datapoints for each of 12 populations. To determine fitness of evolved populations relative to ancestor in the presence/absence of cheats, an LMER model was employed to account for non-independent datapoints (six replicates per population), assigning ‘population’ as a random factor and both condition (treatment/control) and cheats (present/absent) as fixed explanatory factors (including interaction). A general linear model (GLM) was used to investigate whether pyocyanin production is affected by evolution condition (control/treatment lines). The relationship between the proportion of novel morphotypes and *per capita* production of pyoverdine and pyocyanin was examined using separate generalized linear models with a quasi-binomial error structure. Finally, the effect of exogenous pyocyanin on growth rate was calculated as the change in growth relative to the control at each pyocyanin concentration using selection coefficient (*r*): *m*(strain *x*) − mean(*m*(strain *y*)). Using a GLM, the effect of pyocyanin concentration (continuous numeric variable) and strain identity (cooperator or cheat) on promoting growth was investigated (including the interaction). To ensure the effect of adding 10 µM pyocyanin was accounted for, the control treatment relative selection coefficient was included and standardized to zero.

## 3. Results

### (a) Costs and benefits of siderophore production

As with previous studies in iron-limited minimal media [30,44], monocultures of cooperators exhibited a higher growth rate compared with cheat monocultures (Wilcoxon's rank-sum test: *W* = 34, *p* < 0.05); however, this effect was reversed in 1 : 1 co-culture, where cheats had a growth rate advantage over cooperators (one-sample *t*-test of relative fitness against 1: *t*_5_ = 2.74, *p* < 0.05; electronic supplementary material, figure SA2). Thus, while siderophore production carries a group-level fitness benefit is individually costly in this context.

### (b) Quantifying fitness against ancestor and productivity of evolved populations

**Figure 1.**
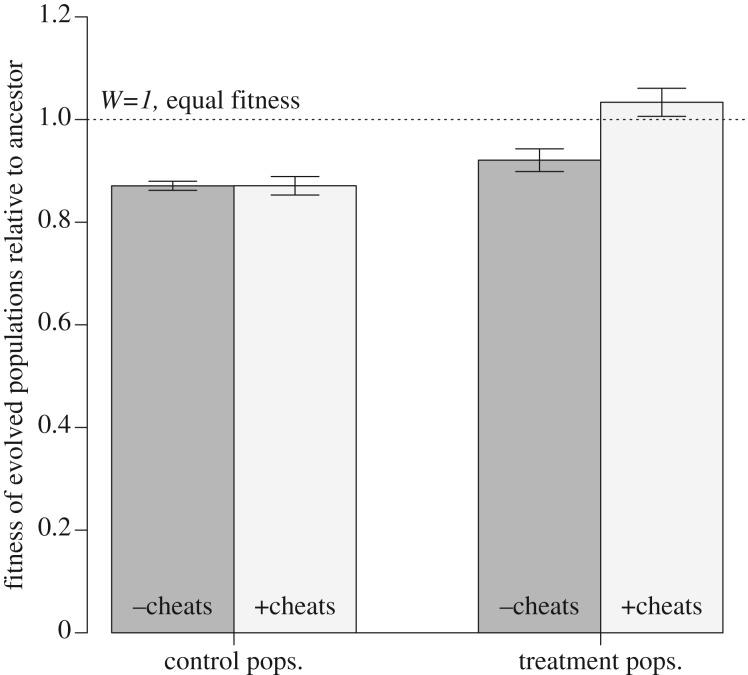
Relative fitness of evolved populations in a 1 : 1 co-culture with ancestral wild-type PAO1, in both the presence and absence of cheats, in iron-limited KB media. Evolved populations were generally less fit than their ancestor, with the exception of treatment populations when competed under the same conditions as which they had evolved in (in the presence of 90% cheats). (LMER treatment × cheat interaction: 
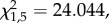
 *p* < 0.0001). Data are means of six replicates per each of 12 evolved populations ± s.e.m.

To determine adaptation to cheats, we competed evolved lines against the ancestor in the presence and absence of 90% cheats: the same conditions as the selective environments. Populations evolved with cheats had a higher fitness than populations evolved in the absence of cheats, but only when cheats were present (LMER treatment × cheat interaction; 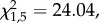 *p* < 0.0001; figure 1).

Given that our experimental design also selected for high within-population yields, we measured the mean growth rate of evolved populations as monocultures under the selective (iron-limited) conditions, finding no difference in the mean growth rate between control and treatment lines (Student's *t*-test, *t*_10_ = 0.03, *p* = 0.98; electronic supplementary material, figure SA3). Finally, we verified that the use of an ancestor possessing a *lacZ* genetic marker in this experiment did not alter relative fitness: in direct 1 : 1 competition with non-*lacZ* ancestor, *W*(*lacZ*) did not differ from 1 (one-sample *t*-test (alt = 1), *t*_5_ = −1.57, *p* *=* 0.18).

### (c) Public goods production following evolution

**Figure 2.**
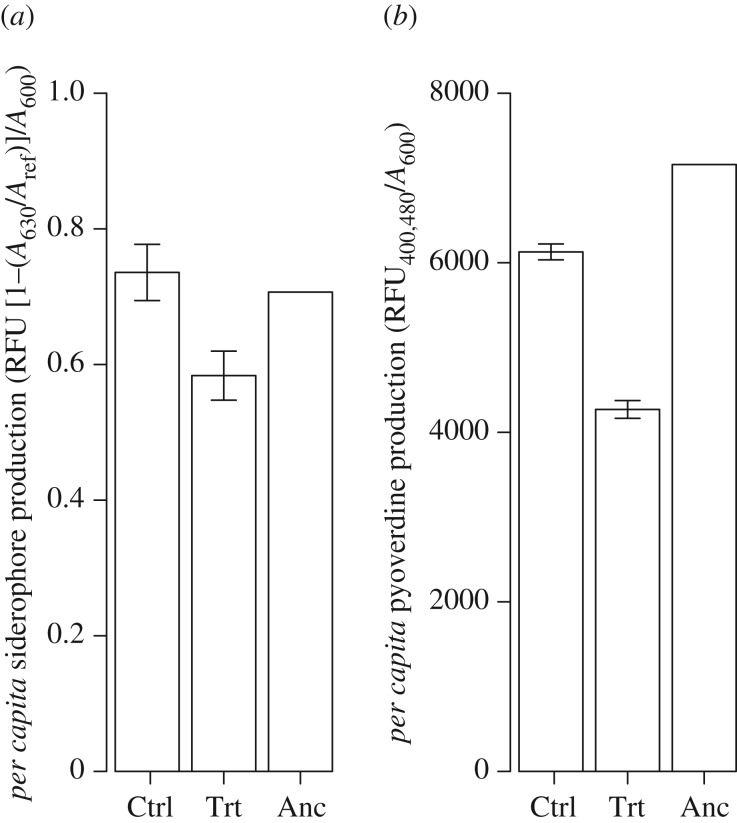
Total siderophore (*a*) and pyoverdine (*b*) production by evolved control populations (Ctrl), treatment populations (Trt; evolved in the presence of 90% cheats) and the ancestral clonal PAO1 (Anc). After approximately 190 generations, treatment populations exhibited reduced *per capita* total siderophore production (one-sample *t*-test (alt = 0.70672), *t*_5_ = 3.4056, *p* < 0.05) and pyoverdine production (one-sided Wilcoxon signed-rank test, alternative = 7876.512, *V* = 4, *p* < 0.0001), relative to the ancestor. For (*a*), data are means of six evolved populations for each treatment ± s.e.m, and the single population of ancestral PAO1. For (*b*), data are means of pyoverdine production for 30 colonies for each evolved population (six evolved populations each for control and treatment condition), and the single ancestral PAO1.

After approximately 190 generations of growth, populations grown in the presence of cheats exhibited reduced *per capita* total siderophore production compared with ancestor (one-sample *t*-test (alt = 0.7), *t*_5_ = 3.41, *p* < 0.05; figure 2*a*) and cheat-free control populations (Student's *t*-test, *t*_10_ = 2.77, *p* < 0.05; figure 2*a*). Testing for pyoverdine specifically, treatment populations showed decreased pyoverdine output compared with ancestor (one-sided Wilcoxon signed-rank test, alternative = 7876.51, *V* = 4, *p* < 0.0001; figure 2*b*) and control populations (LMER: 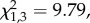 *p* < 0.01; figure 2*b*). Control populations did not differ from ancestral total *per capita* siderophore production (one-sample *t*-test (alt = 0.7) *t*_5_ *=* 0.7, *p* = 0.51; figure 2*a*), but *per capita* pyoverdine output was reduced over the course of the experiment (one-sample *t*-test, alternative = 7876.51, *t*_179_ = 18.63, *p* < 0.0001; figure 2*b*).

### (d) Novel morphotypes

We recorded the appearance of a novel morphotype with a slightly raised surface and reduced surface area in evolved populations (electronic supplementary material, figure SA4 and table SA1). After approximately 190 generations, not only was the frequency of this morphotype significantly higher in our treatment than control populations (Kolmogorov–Smirnov test, *D* = 0.83, *p* < 0.05), it went to fixation in three out of six treatment populations (electronic supplementary material, table SA1). The proportion of evolved novel morphotypes within a population was positively correlated with pyocyanin production (GLM, *F*_1,10_ = 21.22, *p* < 0.001; electronic supplementary material, table SA1), and negatively associated with pyoverdine production (GLM, *F*_1,10_ = 35.64, *p* < 0.001; electronic supplementary material, table SA1). Notably, population-level pyocyanin production was only elevated relative to control when the proportion of novel morphotypes in a population reached fixation, and this variation between populations meant that we did not detect significant differences in total pyocyanin between populations (GLM, *F*_1,10_ = 3.03*, p* *=* 0.11; electronic supplementary material, table SA1). We confirmed that the novel morphotype showed elevated pyocyanin and reduced pyoverdine production relative to ancestral-like colonies, by performing individual assays (replicated thrice) on the eight colonies subsequently selected for sequencing (see below) (pyoverdine: LMER, 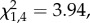 *p* < 0.05; pyocyanin: LMER, 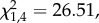 *p* < 0.0001).

To identify the mutation(s) that might confer this wrinkly pyocyanin-overproducing phenotype, the genomes of the ancestral, four smooth (isolated from treatment populations T1, T6 and T3) and four novel morphotypes (isolated from treatment populations T2, T4, T5 and T6) were sequenced and subjected to comparative genomic analysis. We observed deletions of 38.33 kb (on average) in the novel phenotypes compare to the smooth and ancestral strains (electronic supplementary material, figure SA5 and tables SA2 and SA3). Bioinformatic analysis revealed that the deleted genomic fragments contain 19 genes common to all novel morphotypes, including *lasR* and *rsaL* genes encoding a regulator and a repressor of quorum sensing (QS)-regulated factors, respectively (electronic supplementary material, table SA3). These genes were not mutated in any sequenced smooth colonies. Among the four smooth colonies, three in-frame deletions were observed in three different colonies: in *fha1* (2/3) and *ftsY* (1/3)—neither of which are associated with iron-acquisition.

### (e) Addition of exogenous pyocyanin

**Figure 3.**
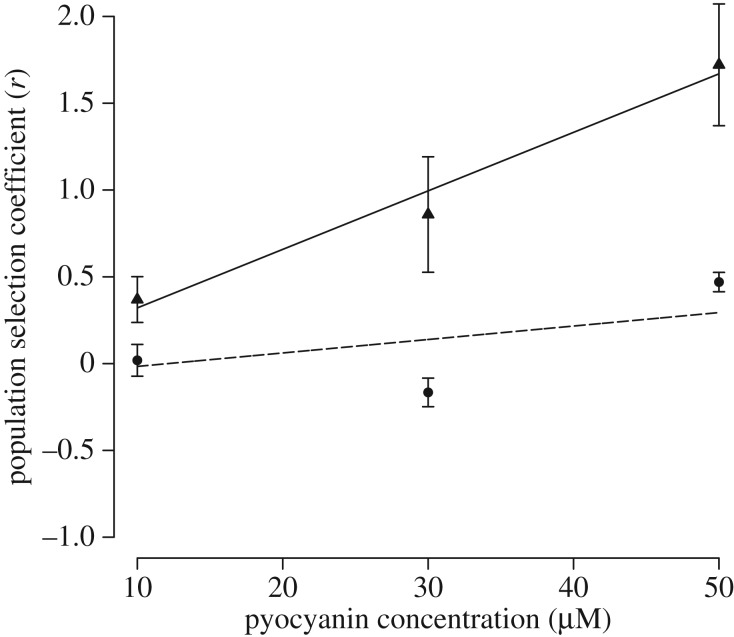
Effect of the addition of exogenous pyocyanin to PAO1 (cooperator; circles and dashed line) and PAO1*ΔpvdDΔpchEF* (cheat; triangles and solid line) populations. Selection coefficient is calculated relative to a control cooperator or cheat population to which no pyocyanin was added. The effect increasing pyocyanin has on relative fitness (*r*) is greatest in cheat populations (GLM, strain identity × pyocyanin concentration, *F*_1,44_ = 12.018, *p* = 0.001). Fitness (*r*) relative to control population (no pyocyanin added) was calculated as *m*(strain *x*) − mean(*m*(strain *y*)). Lines are plotted based on predictions from minimal GLM model. Data are means of six replicates per treatment ± s.e.m.

The addition of exogenous pyocyanin was beneficial for both siderophore-producing and non-producing strains; however, the effect of increasing pyocyanin concentration on growth rate was greatest for non-producing strains (GLM, strain identity × pyocyanin concentration interaction, *F*_1,44_ = 12.02*, p* = 0.001; figure 3).

## 4. Discussion

Here, we investigated whether populations of the bacterium *P. aeruginosa* under selection for high productivity were capable of adapting to the presence of public goods cheats, where public goods in this case are iron-scavenging siderophores. We found that after approximately 190 generations, cheat-adapted populations manifested greater fitness in the presence of cheats compared with control populations, while displaying no apparent growth rate cost when grown in the absence of cheats. While evolved populations had significantly reduced pyoverdine production relative to the ancestor (adaptation to growth conditions), cheat-adapted populations further reduced their production of pyoverdine compared with control populations. Novel morphotypes appeared in five of six cheat-adapted populations, characterized by deletions in *lasR* and *rsaL* genes. The morphotype showed elevated pyocyanin and reduced siderophore production, and its presence resulted in population-level reductions in pyoverdine, while fixation of the morphotype in three of six cheat-adapted populations resulted in increased pyocyanin production. Taken together, these data suggest that one way cooperators may have adapted at least in part to the presence of siderophore cheats is by downregulating siderophore production while upregulating an alternative means to obtain iron (pyocyanin).

Our work has some parallels with recent work by Kümmerli *et al.* [29] whereby coevolution between *P. aeruginosa* siderophore cooperators and cheats drove reduced pyoverdine output by cooperators and blockage of the costly pvdS signalling pathway by coevolving cheats. However, the evolution of reduced pyoverdine in this study and that observed by Kummerli *et al.* is likely to have been driven by different selection pressures. Notably, Kummerli *et al.* performed experimental coevolution by transferring 1% of each culture to new media daily. This design facilitates local competition and low relatedness, which reduces pyoverdine production *per se,* because cheating is favoured [30]. Conversely, the metapopulation design established in our study ensures that any reduction in cooperative output is a direct consequence of adapting to resist the impact of cheats rather than selection for cheating. In line with this, Harrison [28] found that coevolving *P. aeruginosa* cooperators and cheats in metapopulations that impose selection for cooperation reduced pyoverdine production. However, in this case, consequences of lowered pyoverdine for cooperator fitness were not determined, and the coevolutionary design made it difficult to disentangle the effects of altered relatedness and cheating [45].

We have speculated that reductions in pyoverdine and elevated pyocyanin in the novel morphotype may have contributed to the observed adaptation to siderophore cheats in some populations. Reduced pyoverdine production will presumably mitigate some of the fitness costs imposed by pyoverdine cheats [37]. The resulting reduction in iron acquisition may be compensated for by upregulation of pyocyanin, whose canonical function is a toxin [46]. Pyocyanin is a potent reducing agent that converts insoluble ferric (Fe^3+^) to ferrous (Fe^2+^) iron [41], which can diffuse into cells via cell-surface porins (eliminating the requirement of a siderophore-specific receptor). Accordingly, we found that the addition of exogenous pyocyanin had a stronger effect in enhancing growth of siderophore-negative cheats compared with cooperators, suggesting it can compensate for lack of siderophore production. Moreover, recent studies investigating adaptation of *P. aeruginosa* to the antimicrobial gallium nitrate show that cells become resistant by downregulating pyoverdine (which acts in this case as a gallium transporter) and upregulating pyocyanin [47,48]. However, while populations with high frequencies of novel morphotypes may have benefited from this increase in pyocyanin, the remaining populations are likely to have evolved alternative, unknown strategies to cope with reduced pyoverdine production. One possibility is that prudent regulation of cooperative traits can impede the spread of cheats, by only cooperating when the costs of doing so are minimal. For example, the diffusible *P. aeruginosa* carbon-rich rhamnolipid is expressed only when growth is limited by another nutrient source [49]. However, in our experiment, the costs of cooperating were consistently high, based on our finding that cheats invaded cooperators when competed at 1 : 1.

The novel morphotype characterized by reduced pyoverdine and increased pyocyanin production carried deletions in *lasR* and *rsaL* genes. LasR and RsaL are two transcriptional regulators that positively and negatively regulate the expression of QS-regulated virulence factors, respectively [50–52]. Pyoverdine production is under positive control of the Las system and its inactivation has been reported to reduce the production of this siderophore [53]. LasR also regulates pyocyanin production; however, the pyocyanin biosynthetic operon *phzA*-*G1* is under direct repression of RsaL, and it has been shown that cells which lack *rsaL* overexpress pyocyanin [54]. It therefore seems likely that these deletions play a role in the observed phenotypic changes in the novel morphotype, although we cannot rule out that other gene deletions in this novel morphotype may have contributed to changes in siderophore and pyocyanin production, as well as adaptation more generally.

Our finding that the growth rate of siderophore cheats in iron-limited media can be rescued by the addition of exogenous pyocyanin suggests that like siderophores, pyocyanin may also act as a public good. This is further supported by studies in animal models demonstrating reduced growth and virulence of pyocyanin negative mutants compared with wild-type [55,56], but that mutant growth is enhanced by the presence of wild-type producers in mixed infections, or the addition of exogenous pyocyanin [56]. Note that in this case, pyocyanin is probably not linked to iron-scavenging, as non-producers had intact siderophores, and pyocyanin has a range of additional *in vivo* activities such as apoptosis of neutrophils that could enhance growth. This then begs the question: why were not pyocyanin overproducers in our evolved cheat-adapted populations exploited by individuals making less pyocyanin? The most likely explanation is that pyocyanin over-production imposes a small metabolic cost (relative to siderophores), at least under these experimental conditions; hence, pyocyanin non-producers would have little, if any, fitness advantage. However, our results do not rule out the possibility that pyocyanin producers could ultimately be exploited by the evolution of pyocyanin cheats in this or other environments.

One counterintuitive result was the loss of fitness through time in evolved populations: competing ancestral against evolved cooperator populations in the presence and absence of cheats demonstrated that evolved control populations were consistently outcompeted by the ancestor, while evolved treatment populations managed to negate this only in the presence of cheats. The inherent disadvantage of evolved populations could not be attributed to differences in growth between PAO1^R^*lacZ* and PAO1^R^, and was potentially a consequence of population bottlenecking resulting from transfer of single clones, which may have resulted in the fixation of deleterious mutations [57].

It is always debatable whether *in vitro* results are relevant to the real world. While siderophore mutants are present in natural populations [58,59] and can have a selective advantage when rare [15], it is unclear if (i) they act as cheats in this context and (ii) selection has acted in ways that mitigate their exploitation, as observed here. Pyocyanin over-producing genotypes are associated with exacerbated cystic fibrosis infections [60] and while it can be speculated that these phenotypes represent an alternative mechanism of iron-acquisition, these correlational data can of course be open to different interpretations. Nonetheless, the evolutionary impact of altered social interactions between microbes should be carefully considered in all cases, particularly in light of the development of novel therapeutics aimed at disrupting microbial social interactions [56,61]. Given that pyocyanin directly harms host cells, kills competitors and results in more virulent infections [41,46], investigation into whether pyocyanin contributes significantly as an iron-uptake mechanism in natural populations is warranted.

## Supplementary Material

Appendix A1

## Supplementary Material

Appendix A2

## Supplementary Material

Data

## Data accessibility

The datasets supporting this article have been uploaded as part of the electronic supplementary material.
