# Supplementary material for: Adaptation to public goods cheats in Pseudomonas aeruginosa
Source: Proc Biol Sci. 2017 Jul 26;284(1859):20171089. doi: 10.1098/rspb.2017.1089 (PMC5543229; doi:10.1098/rspb.2017.1089)
Supplement: Appendix A1 [file rspb20171089supp1.pdf]

Appendix A1

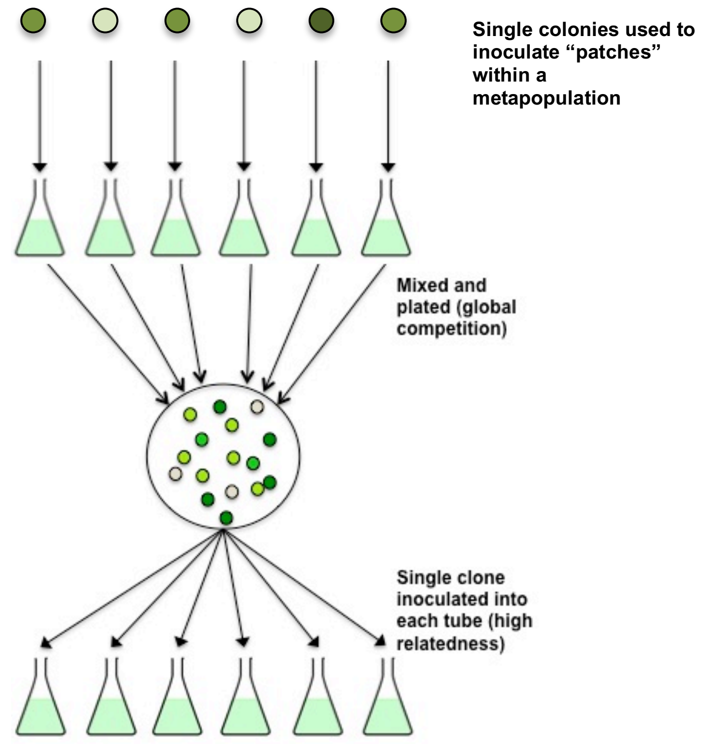

Figure A1: Experimental design. We imposed global competition between subpopulations by periodically mixing equal quantities of each culture and plating 50μl on KB agar. Six single colonies were selected and each used to inoculate new culture, so that each subpopulation consisted of a single clone (high relatedness).

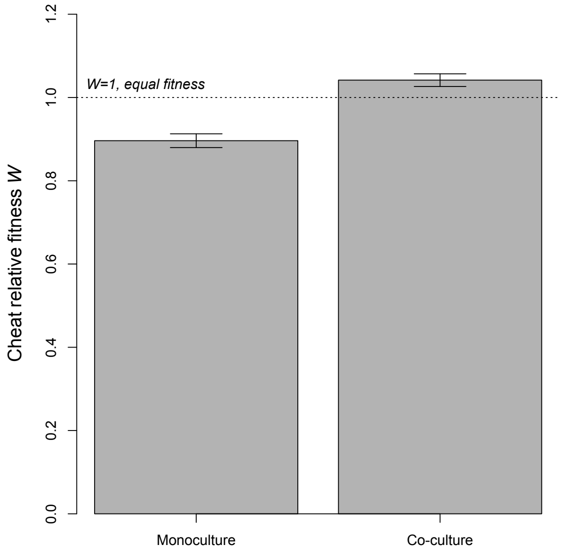

Figure A2: Fitness (*W*) of siderophore-negative cheat relative to producing cooperator in an iron-limited environment. In monoculture, cheats exhibit poor growth relative to cooperators as they cannot gain access to iron using siderophores (*W*(cheat) < 1 (Wilcoxon signed rank test: *V*=0, *p*<0.05). In co-culture, cheats invade populations as they take advantage of siderophores produced by cooperators (*W*(cheat) > 1 (1-sample t-test: t_5=2.743, *p*<0.05). Each *W*(cheat) datapoint in monoculture is calculated as *m*(cheat)/mean(*m*(cooperators)), and in co-culture as *m*(cheat)/(*m*(cooperator). Data are means of 6 populations for each treatment ± SEM

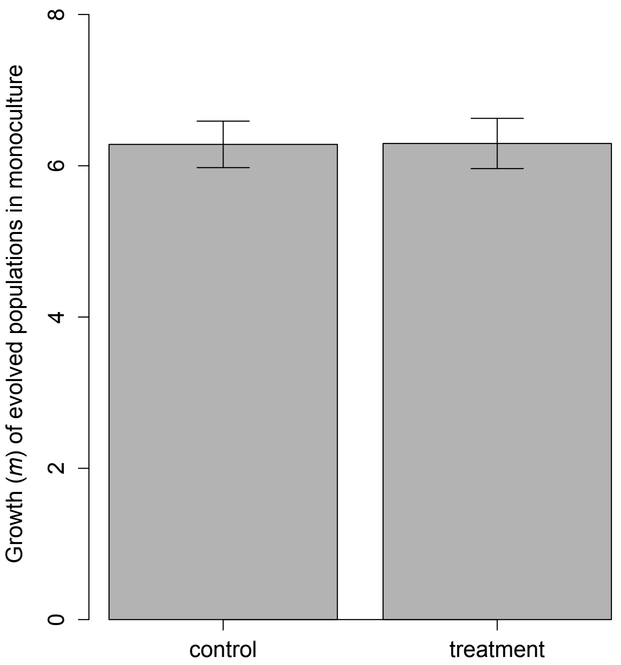

Figure A3: Evolved populations grown as monocultures in iron-limited KB broth. Population growth (m) is calculated as ln(final density/start density). We find no difference in population growth between control and treatment lines (Student's t-test: t_{10}= 0.0268, p=0.98). Data are means of 6 populations for each treatment ± SEM.

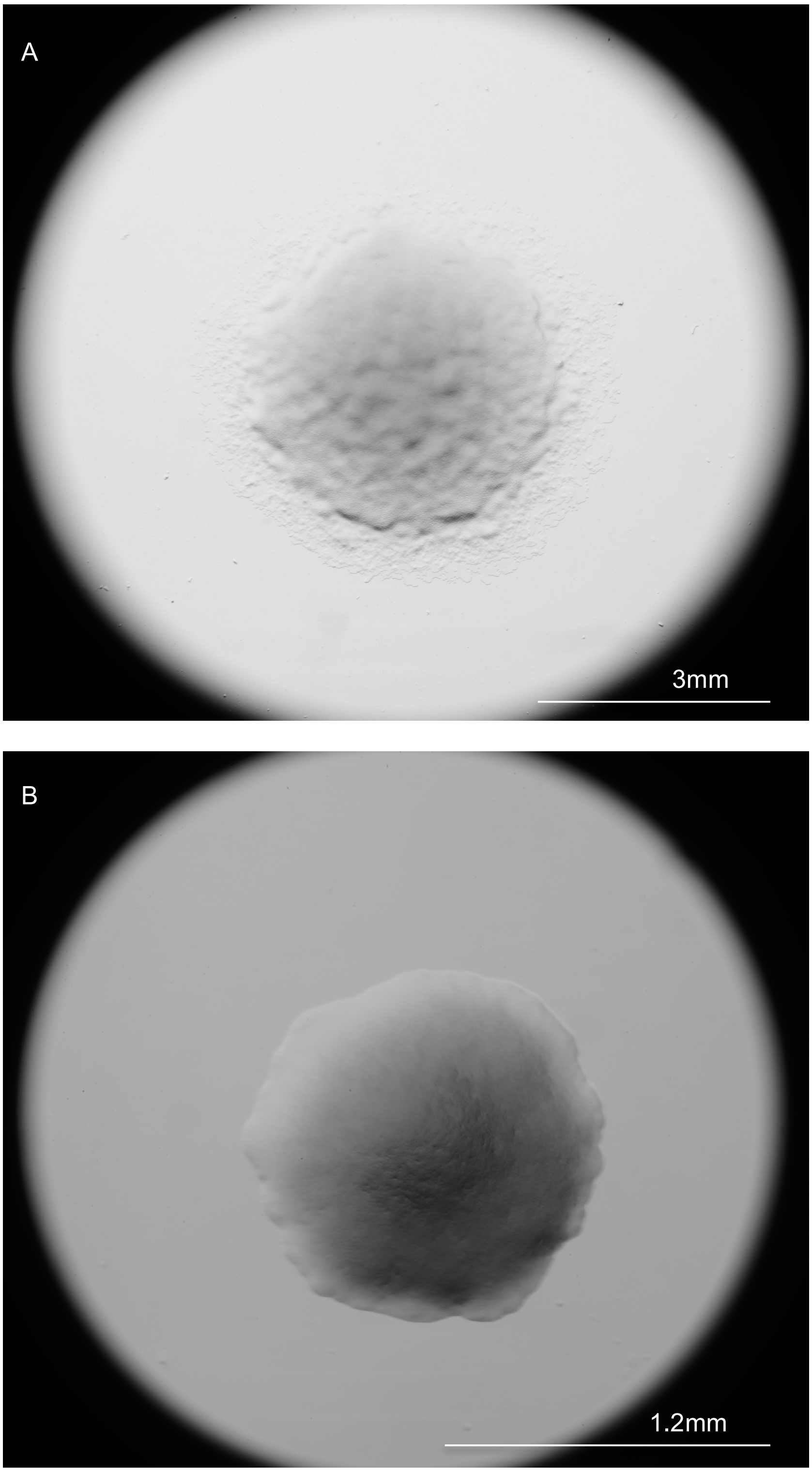

Figure A4: A: smooth wildtype PAO1 colony (3X magnification). **B**: novel morphotype observed in 5 out of 6 cheat-adapted populations (8X magnification).

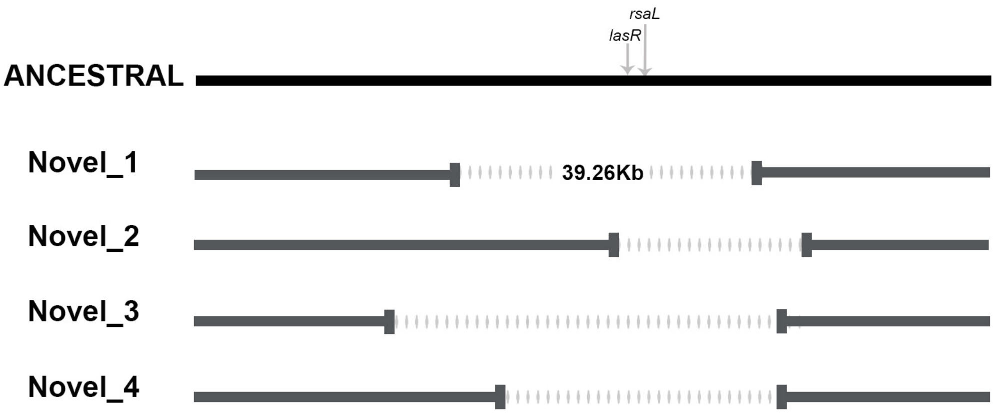

Figure A5: Genomic diagram of ancestral PAO1 strain and independent novel morphotypes clones evolved in the presence of cheats (90%).

Table A1

Proportion of evolved novel morphotypes and corresponding mean *per capita* pyoverdine and pyocyanin output

Population	Novel morphotypes (Proportion)	Relative pyoverdine production ± s.e. RFU($_{400,460}$/A_{600})	Population relative pyocyanin production A_{691}/ A_{600}
T1	0.000	5782.301 ± 182.696	0.063
T2	1.000	3158.189 ± 163.098	0.206
T3	0.062	5439.163 ± 239.419	0.033
T4	1.000	3460.821 ± 170.651	0.151
T5	1.000	3569.158 ± 172.089	0.355
T6	0.229	4211.073 ± 150.020	0.093
C1	0.007	6020.19 ± 225.34	0.074
C2	0.014	6068.807 ± 172.659	0.051
C3	0.000	4942.095 ± 237.019	0.031
C4	0.000	6575.874 ±193.413	0.088
C5	0.000	6317.303 ±169.590	0.074
C6	0.000	6844.052 ± 220.166	0.074

Note. Population identity is assigned "T" for treatment populations (evolved in presence of cheats) and "C" for control populations (evolved in absence of cheats). Pyoverdine and pyocyanin values have been standardised by population density as measured by A_{600}. Pyoverdine production is mean ± se of 30 random colonies isolated per population.

Table A2

Mutations acquired by smooth and novel morphotypes in the presence of cheats (90%) identified by whole genome re-sequencing.

Clone	Population	Start Position	End Position	Mutation	Annotation
Smooth_1	T3	98901	98913	inframe deletion ΔCAGCCACAGCCA	*fha1*; ΔQ282_P285
Smooth_2	T3	98901	98913	inframe deletion ΔCAGCCACAGCCA	*fha1*; ΔQ282_P285
Soooth_3	T6	417866	417872	inframe deletion ΔAGCCCG	*ftsY*; ΔE129_P130
Novel_1	T2	3597114	3597114	synonymous G>A	PA3206; P391P
		1536198	1575459	del 39.26Kb	ΔPA1411-PA1447
Novel_2	T4	1556526	1581415	del 24.89Kb	ΔPA1429-PA1452
Novel_3	T5	1526604	1578142	del 51.54Kb	ΔPA1404-PA1450
Novel_4	T6	417866	417872	inframe deletion ΔAGCCCG	*ftsY*; ΔE129_P130
		1540493	1578152	del 37.66Kb	ΔPA1416-PA1450

Table A3

Genes affected by large 24-39Kb deletion in novel morphotypes. Genes in bold are common to all four novel strains.

Colony	Annotation	Function	Confidence	Position
**Novel 1**	PA1411	hypothetical protein	class 4	1535586 - 1536497
	PA1412	hypothetical protein	class 4	1536457 - 1537650
	PA1413	probable transcriptional regulator	class 3	1537755 - 1538627
	PA1414	hypothetical protein	class 4	1538683 - 1538916
	PA1415	hypothetical protein	class 4	1538976 - 1539704
	PA1416	conserved hypothetical protein	class 4	1539749 - 1541131
	PA1417	probable decarboxylase	class 3	1541145 - 1542746
	PA1418	probable sodium:solute symport protein	class 3	1542822 - 1544213
	PA1419	probable transporter	class 3	1544307 - 1545818
	PA1420	hypothetical protein	class 4	1545837 - 1546256
	gbuA	guanidinobutyrase	class 1	1546271 - 1547230
	GbuR	transcription activator of gbuA;	class 1	1547437 - 1548330
	bdlA	Biofilm dispersion protein	class 1	1548334 - 1549587
	PA1424	hypothetical protein	class 4	1549883 - 1550494
	PA1425	probable ATP-binding component of ABC transporter	class 3	1550984 - 1552600
	PA1426	hypothetical protein	class 4	1552641 - 1552997
	PA1427	hypothetical protein	class 4	1553112 - 1553675
	PA1428	conserved hypothetical protein	class 4	1554415 - 1554846
	**PA1429**	**probable cation-transporting P-type ATPase**	**class 3**	**1555012 - 1557720**
	**lasR**	**transcriptional regulator LasR**	**class 1**	**1558171 - 1558890**
	**rsaL**	**regulatory protein RsaL**	**class 1**	**1558880 - 1559122**
	**LasI**	**autoinducer synthesis protein LasI**	**class 1**	**1559254 - 1559859**
	**PA1433**	**conserved hypothetical protein**	**class 4**	**1559966 - 1561918**
	**PA1434**	**hypothetical protein**	**class 4**	**1561919 - 1562632**
	**PA1435**	**probable Resistance-Nodulation-Cell Division (RND) efflux membrane fusion protein precursor**	**class 3**	**1562813 - 1563970**
	**PA1436**	**probable Resistance-Nodulation-Cell Division (RND) efflux transporter**	**class 3**	**1563967 - 1567077**
	**PA1437**	**probable two-component response regulator**	**class 3**	**1567181 - 1567870**
	**PA1438**	**probable two-component sensor**	**class 3**	**1567848 - 1569293**
	**PA1439**	**conserved hypothetical protein**	**class 4**	**1569302 - 1569709**
	**PA1440**	**hypothetical protein**	**class 4**	**1569721 - 1570308**
	**PA1441**	**putative flagellar hook-length control protein FliK**	**class 2**	**1570496 - 1571779**
	**PA1442**	**conserved hypothetical protein**	**class 4**	**1572023 - 1572544**
	**FliM**	**flagellar motor switch protein FliM**	**class 2**	**1572552 - 1573523**
	**FliN**	**flagellar motor switch protein FliN**	**class 1**	**1573551 - 1574024**
	**FliO**	**flagellar protein FliO**	**class 1**	**1574026 - 1574478**
	**FliP**	**flagellar biosynthetic protein FliP**	**class 2**	**1574475 - 1575242**
	**FliQ**	**flagellar biosynthetic protein FliQ**	**class 2**	**1575290 - 1575559**

**Novel 2**	**PA1429**	**probable cation-transporting P-type ATPase**	**class 3**	**1555012 - 1557720**
	**lasR**	**transcriptional regulator LasR**	**class 1**	**1558171 - 1558890**
	**rsaL**	**regulatory protein RsaL**	**class 1**	**1558880 - 1559122**
	**LasI**	**autoinducer synthesis protein LasI**	**class 1**	**1559254 - 1559859**
	**PA1433**	**conserved hypothetical protein**	**class 4**	**1559966 - 1561918**
	**PA1434**	**hypothetical protein**	**class 4**	**1561919 - 1562632**
	**PA1435**	**probable Resistance-Nodulation-Cell Division (RND) efflux membrane fusion protein precursor**	**class 3**	**1562813 - 1563970**
	**PA1436**	**probable Resistance-Nodulation-Cell Division (RND) efflux transporter**	**class 3**	**1563967 - 1567077**
	**PA1437**	**probable two-component response regulator**	**class 3**	**1567181 - 1567870**
	**PA1438**	**probable two-component sensor**	**class 3**	**1567848 - 1569293**
	**PA1439**	**conserved hypothetical protein**	**class 4**	**1569302 - 1569709**
	**PA1440**	**hypothetical protein**	**class 4**	**1569721 - 1570308**
	**PA1441**	**putative flagellar hook-length control protein FliK**	**class 2**	**1570496 - 1571779**
	**PA1442**	**conserved hypothetical protein**	**class 4**	**1572023 - 1572544**
	**FliM**	**flagellar motor switch protein FliM**	**class 2**	**1572552 - 1573523**
	**FliN**	**flagellar motor switch protein FliN**	**class 1**	**1573551 - 1574024**
	**FliO**	**flagellar protein FliO**	**class 1**	**1574026 - 1574478**
	**FliP**	**flagellar biosynthetic protein FliP**	**class 2**	**1574475 - 1575242**
	**FliQ**	**flagellar biosynthetic protein FliQ**	**class 2**	**1575290 - 1575559**
	FliR	flagellar biosynthetic protein FliR	class 2	1575559 - 1576335
	FlhB	flagellar biosynthetic protein FlhB	class 2	1576338 - 1577474
	PA1450	conserved hypothetical protein	class 4	1577547 - 1578806
	PA1451	conserved hypothetical protein	class 4	1578839 - 1580182
	flhA	flagellar biosynthesis protein FlhA	class 2	1580321 - 1582444
**novel 3**	PA1404	hypothetical protein	class 4	1526430 - 1526657
	PA1405	probable helicase	class 3	1527191 - 1528783
	PA1406	hypothetical protein	class 4	1528803 - 1529561
	PA1407	hypothetical protein	class 4	1529597 - 1530538
	PA1408	hypothetical protein	class 4	1530605 - 1533028
	aphA	acetylpolyamine aminohydrolase	class 2	1533238 - 1534278
	PA1410	probable periplasmic spermidine/putrescine-binding protein	class 3	1534289 - 1535380
	PA1411	hypothetical protein	class 4	1535586 - 1536497
	PA1411	hypothetical protein	class 4	1535586 - 1536497
	PA1412	hypothetical protein	class 4	1536457 - 1537650
	PA1413	probable transcriptional regulator	class 3	1537755 - 1538627
	PA1414	hypothetical protein	class 4	1538683 - 1538916
	PA1415	hypothetical protein	class 4	1538976 - 1539704
	PA1416	conserved hypothetical protein	class 4	1539749 - 1541131
	PA1417	probable decarboxylase	class 3	1541145 - 1542746
	PA1418	probable sodium:solute symport protein	class 3	1542822 - 1544213
	PA1419	probable transporter	class 3	1544307 - 1545818
	PA1420	hypothetical protein	class 4	1545837 - 1546256
	gbuA	guanidinobutyrase	class 1	1546271 - 1547230

	GbuR	transcription activator of gbuA	class 1	1547437 - 1548330
	bdlA	Biofilm dispersion protein	class 1	1548334 - 1549587
	PA1424	hypothetical protein	class 4	1549883 - 1550494
	PA1425	probable ATP-binding component of ABC transporter	class 3	1550984 - 1552600
	PA1426	hypothetical protein	class 4	1552641 - 1552997
	PA1427	hypothetical protein	class 4	1553112 - 1553675
	PA1428	conserved hypothetical protein	class 4	1554415 - 1554846
	**PA1429**	**probable cation-transporting P-type ATPase**	**class 3**	**1555012 - 1557720**
	**lasR**	**transcriptional regulator LasR**	**class 1**	**1558171 - 1558890**
	**rsaL**	**regulatory protein RsaL**	**class 1**	**1558880 - 1559122**
	**LasI**	**autoinducer synthesis protein LasI**	**class 1**	**1559254 - 1559859**
	**PA1433**	**conserved hypothetical protein**	**class 4**	**1559966 - 1561918**
	**PA1434**	**hypothetical protein**	**class 4**	**1561919 - 1562632**
	**PA1435**	**probable Resistance-Nodulation-Cell Division (RND) efflux membrane fusion protein precursor**	**class 3**	**1562813 - 1563970**
	**PA1436**	**probable Resistance-Nodulation-Cell Division (RND) efflux transporter**	**class 3**	**1563967 - 1567077**
	**PA1437**	**probable two-component response regulator**	**class 3**	**1567181 - 1567870**
	**PA1438**	**probable two-component sensor**	**class 3**	**1567848 - 1569293**
	**PA1439**	**conserved hypothetical protein**	**class 4**	**1569302 - 1569709**
	**PA1440**	**hypothetical protein**	**class 4**	**1569721 - 1570308**
	**PA1441**	**putative flagellar hook-length control protein FliK**	**class 2**	**1570496 - 1571779**
	**PA1442**	**conserved hypothetical protein**	**class 4**	**1572023 - 1572544**
	**FliM**	**flagellar motor switch protein FliM**	**class 2**	**1572552 - 1573523**
	**FliN**	**flagellar motor switch protein FliN**	**class 1**	**1573551 - 1574024**
	**FliO**	**flagellar protein FliO**	**class 1**	**1574026 - 1574478**
	**FliP**	**flagellar biosynthetic protein FliP**	**class 2**	**1574475 - 1575242**
	**FliQ**	**flagellar biosynthetic protein FliQ**	**class 2**	**1575290 - 1575559**
	FliR	flagellar biosynthetic protein FliR	class 2	1575559 - 1576335
	FlhB	flagellar biosynthetic protein FlhB	class 2	1576338 - 1577474
	PA1450	conserved hypothetical protein	class 4	1577547 - 1578806
**novel 4**	PA1416	conserved hypothetical protein	class 4	1539749 - 1541131
	PA1417	probable decarboxylase	class 3	1541145 - 1542746
	PA1418	probable sodium:solute symport protein	class 3	1542822 - 1544213
	PA1419	probable transporter	class 3	1544307 - 1545818
	PA1420	hypothetical protein	class 4	1545837 - 1546256
	gbuA	guanidinobutyrase	class 1	1546271 - 1547230
	GbuR	guanidinobutyrase	class 1	1547437 - 1548330
	bdlA	transcription activator of gbuA	class 1	1548334 - 1549587
	PA1424	hypothetical protein	class 4	1549883 - 1550494
	PA1425	probable ATP-binding component of ABC transporter	class 3	1550984 - 1552600
	PA1426	hypothetical protein	class 4	1552641 - 1552997
	PA1427	hypothetical protein	class 4	1553112 - 1553675
	PA1428	conserved hypothetical protein	class 4	1554415 - 1554846
	**PA1429**	**probable cation-transporting P-type ATPase**	**class 3**	**1555012 - 1557720**
	**lasR**	**transcriptional regulator LasR**	**class 1**	**1558171 - 1558890**

**rsaL**	**regulatory protein RsaL**	**class 1**	**1558880 - 1559122**
**LasI**	**autoinducer synthesis protein LasI**	**class 1**	**1559254 - 1559859**
**PA1433**	**conserved hypothetical protein**	**class 4**	**1559966 - 1561918**
**PA1434**	**hypothetical protein**	**class 4**	**1561919 - 1562632**
**PA1435**	**probable Resistance-Nodulation-Cell Division (RND) efflux membrane fusion protein precursor**	**class 3**	**1562813 - 1563970**
**PA1436**	**probable Resistance-Nodulation-Cell Division (RND) efflux transporter**	**class 3**	**1563967 - 1567077**
**PA1437**	**probable two-component response regulator**	**class 3**	**1567181 - 1567870**
**PA1438**	**probable two-component sensor**	**class 3**	**1567848 - 1569293**
**PA1439**	**conserved hypothetical protein**	**class 4**	**1569302 - 1569709**
**PA1440**	**hypothetical protein**	**class 4**	**1569721 - 1570308**
**PA1441**	**putative flagellar hook-length control protein FliK**	**class 2**	**1570496 - 1571779**
**PA1442**	**conserved hypothetical protein**	**class 4**	**1572023 - 1572544**
**FliM**	**flagellar motor switch protein FliM**	**class 2**	**1572552 - 1573523**
**FliN**	**flagellar motor switch protein FliN**	**class 1**	**1573551 - 1574024**
**FliO**	**flagellar protein FliO**	**class 1**	**1574026 - 1574478**
**FliP**	**flagellar biosynthetic protein FliP**	**class 2**	**1574475 - 1575242**
**FliQ**	**flagellar biosynthetic protein FliQ**	**class 2**	**1575290 - 1575559**
FliR	flagellar biosynthetic protein FliR	class 2	1575559 - 1576335
FlhB	flagellar biosynthetic protein FlhB	class 2	1576338 - 1577474
PA1450	conserved hypothetical protein	class 4	1577547 - 1578806
