# Supplementary material for: Adaptation to public goods cheats in Pseudomonas aeruginosa
Source: Proc Biol Sci. 2017 Jul 26;284(1859):20171089. doi: 10.1098/rspb.2017.1089 (PMC5543229; doi:10.1098/rspb.2017.1089)
Supplement: Appendix A2 [file rspb20171089supp2.docx]

**Appendix A2**

*Resequencing methods and bioinformatic analysis.*

Preparation of sequence data was performed as described previously [1]. Briefly, sequenced read data were trimmed using Cutadapt version 1.2.1 [2] setting option -O 3 and Sickle version 1.2 (<https://github.com/najoshi/sickle>) setting minimum window quality score to 20 and omitting reads shorter than 10 bp after trimming but retaining single remaining reads from pairs. The Genome Analysis Toolkit (GATK) [3] Indel Realigner module [4] was used to realign raw reads around indels and duplicate reads were identified and removed with Picard (<https://github.com/broadinstitute/picard>). Single nucleotide polymorphism, insertion, and deletion discovery from the sequence data were performed with GATK's Unified Genotyper module. Standard conservative filtering parameters were used to provide high-quality variant calls [5]. To identify structural variants Delly [6] was run in each mapped BAM files with default parameters.

1. Williams D, Evans B, Haldenby S, Walshaw MJ, Brockhurst MA, Winstanley C, Paterson S. (2015). Divergent, Coexisting Pseudomonas aeruginosa Lineages in Chronic Cystic Fibrosis Lung Infections. *American Journal of Respiratory and Critical Care Medicine* **191**: 775-85. <https://doi.org/10.1164/rccm.201409-1646oc>

2. Martin M. (2011). Cutadapt removes adapter sequences from high-throughput sequencing reads. *EMBnet Journal* **17**:10–12. https://doi.org/10.14806/ej.17.1.200

3. McKenna A, Hanna M, Banks E, Sivachenko A, Cibulskis K, Kernytsky A, Garimella K, Altshuler D, Gabriel S, Daly M, *et al.* (2010). The Genome Analysis Toolkit: A MapReduce framework for analyzing next-generation DNA sequencing data. *Genome Research* **20**: 1297–1303. <https://doi.org/10.1101/gr.107524.110>

4. DePristo MA, Banks E, Poplin R, Garimella KV, Maguire JR, Hartl C, Philippakis AA, del Angel G, Rivas MA, Hanna M, *et al*. (2011). A framework for variation discovery and genotyping using next-generation DNA sequencing data. *Nature Genetics* **43**: 491–8. <https://doi.org/10.1038/ng.806>

5. Van der Auwera GA, Carneiro MO, Hartl C, Poplin R, del Angel G, Levy-Moonshine A, Jordan T, Shakir K, Roazen D, Thibault J, *et al*. (2013). From FastQ data to high confidence variant calls: The Genome Analysis Toolkit best practices pipeline. *Curr Protoc Bioinfor* **43**: 11–33. <https://doi.org/10.1002/0471250953.bi1110s43>

6. Rausch T, Zichner T, Schlattl A, Stutz AM, Benes V, Korbel JO. (2012). DELLY: structural variant discovery by integrated paired-end and split-read analysis. *Bioinformatics* **28**: 333–339. https://doi.org/10.1093/bioinformatics/bts378
